# A Systematic Review of the Retrograde Drilling Approach for Osteochondral Lesion of the Talus: Questioning Surgical Approaches, Outcome Evaluation and Gender-Related Differences

**DOI:** 10.3390/jcm12134523

**Published:** 2023-07-06

**Authors:** Francesca Veronesi, Melania Maglio, Silvia Brogini, Antonio Mazzotti, Elena Artioli, Gianluca Giavaresi

**Affiliations:** 1 Surgical Sciences and Technologies, IRCCS Istituto Ortopedico Rizzoli, Via di Barbiano 1/10, 40136 Bologna, Italy; francesca.veronesi@ior.it (F.V.); silvia.brogini@ior.it (S.B.); gianluca.giavaresi@ior.it (G.G.); 2 1st Orthopaedic and Traumatologic Clinic, IRCCS Istituto Ortopedico Rizzoli, Via G.C.Pupilli 1, 40136 Bologna, Italy; antonio.mazzotti@ior.it (A.M.); elena.artioli@ior.it (E.A.)

**Keywords:** retrograde drilling, osteochondral lesions, clinical studies, review, gender, orthopaedic

## Abstract

Background: Retrograde drilling (RD) is a minimally invasive surgical procedure mainly used for non-displaced osteochondral lesions (OCL) of the talus, dealing with subchondral necrotic sclerotic lesions or subchondral cysts without inducing iatrogenic articular cartilage injury, allowing the revascularization of the subchondral bone and new bone formation. Methods: This systematic review collected and analyzed the clinical studies of the last 10 years of literature, focusing not only on the clinical results but also on patients’ related factors (gender, BMI, age and complications). Results: Sixteen clinical studies were retrieved, and differences in the type of study, follow-up, number and age of patients, lesion type, dimensions, grades and comparison groups were observed, making it difficult to draw conclusions. Nevertheless, lesions on which RD showed the best results were those of I–III grades and not exceeding 150 mm^2^ in size, showing overall positive results, a good rate of patient satisfaction, improvements in clinical scores, pain reduction and return to daily activities and sports. Conclusions: There are still few studies dealing with the issue of post-surgical complications and gender-related responses. Further clinical or preclinical studies are thus mandatory to underline the success of this technique, also in light of gender differences.

## 1. Introduction

The ankle is the most damaged joint of the body because it supports body forces and mass, sustaining the highest weight per unit area compared to all the other joints [1]. 

Osteochondral lesions (OCL) of the talus are the most common injury occurring in the ankle, especially among athletes at all levels, because of ankle sprains and fractures [2]. OCL affects talar articular cartilage and subchondral bone (SB), and worldwide, 50% of patients with ankle sprains and two out of three patients with chronic lateral ankle instability are affected by OCL [3,4]. Talar dome OCL has an incidence of 0.9% among all talar OCL and can be idiopathic or a consequence of ankle trauma, which can be classified as acute (for trauma that occurred 6 weeks before) or chronic (for trauma that occurred earlier) [5,6,7]. Usually, OCL is localized in the posteromedial aspect of the talus and, unlike knee OCL, spreads deeper into SB, causing a higher frequency of subchondral cysts [8]. The common clinical symptoms of OCL are chronic ankle pain, swelling, stiffness, instability, increased fall risk and limited functional activity [9]. 

Regarding management strategies, nonoperative conservative strategies are employed for acute and nondisplaced lesions, while surgical procedures are performed when the lesions are chronic and displaced [10]. More precisely, conservative treatments are indicated for stable lesions with a Berndt–Harty–Loomer (BHL) classification stage ≤ III. Such approaches foresee activity modifications (such as low-impact weight-bearing and immobilization) or intra-articular injections of platelet-rich plasma (PRP) or hyaluronic acid [7]. When conservative treatments fail (for 3–6 months), or in the presence of loose bodies, unstable lesions, SB sclerosis or BHL > III, surgical treatments take over [10]. 

Several different surgical treatments are employed for talus OCL depending on the defect stage and size. Arthroscopic or open surgery techniques primarily aim to revitalize the necrosis of SB. Bone marrow stimulation (BMS) techniques are the most used surgical procedures for the treatment of talus OCL due to their simplicity, low morbidity, low costs and good-to-excellent results. BMS techniques penetrate the SB plate and induce vascular access to SB, forming a clot that fills the defect [11]. This clot is rich in marrow elements, such as mesenchymal stem cells (MSCs), that can differentiate into chondrogenic or osteogenic lineages [12,13]. BMS techniques include abrasion arthroplasty, microfracture or drilling (anterograde and retrograde). The drilling is carried out with a Kirschner wire or a drill bit and through anterograde or retrograde approaches. Unlike microfractures, the drilling technique reaches a deeper part of the subchondral bone, but on the other hand, it induces thermal necrosis [14]. 

The anterograde drilling (AD) approach, also named the transmalleolar approach, enters the medial malleolus through cartilage and, for this reason, it may cause epiphyseal line injury [15]; in addition, dorsomedial talar dome lesions are frequently inaccessible with AD techniques. Retrograde drilling (RD), also named transtalar drilling, was developed as an alternative approach: it exploits drill guides, intraoperative fluoroscopy, or computer-assisted navigation and allows SB area to be revitalized without damaging the overlayed cartilage [16]. 

As first reported by Lee and Mercurio in 1981 [17], RD is minimally invasive and does not induce cartilage and epiphyseal line injuries. It is mainly used for undisplaced talus OCL, dealing with subchondral necrotic sclerotic lesions or subchondral cysts without inducing iatrogenic articular cartilage injury [18]. It is useful when the osteochondral fragment is stable with normal or nearly normal overlaying cartilage, inducing the revascularization of the SB, then leading to new bone formation. Although other surgical techniques are highly recommended for the treatment of OCL; however, RD is indicated when the defect is difficult to reach through the usual arthroscopic portals, showing good results in 80–100% of the patients [19]. In the last 10 years, few well-designed clinical studies in the literature reported the results of RD for treating talus OCL. 

The present review aimed to systematically revise the literature of the last 10 years to collect all the clinical studies that employed RD as surgical treatment for talus OCL, focusing on the clinical results and complications. The main clinical results were included in this systematic review, with particular attention paid to the possible association between the main results and the gender, body mass index (BMI) or age of the patients. 

## 2. Materials and Methods

### 2.1. Eligibility Criteria

To select the relevant papers included in this systematic review, a PICO question [population of interest (P), Intervention (I), comparators and outcomes (CO)] statement was formulated. 

The “Population” considered was represented by randomized, prospective, retrospective, observational clinical studies and case reports involving patients affected by OCL of the talus. The “Intervention” considered was RD procedures with the specific indication of any augmented treatments. The “Comparator” was any reference group. The considered primary outcome was the main clinical results and complications associated with the RD procedures. In addition, a secondary outcome was represented by the correlation between clinical results and patient gender, BMI or age.

### 2.2. Search Strategy

**Figure 1:**
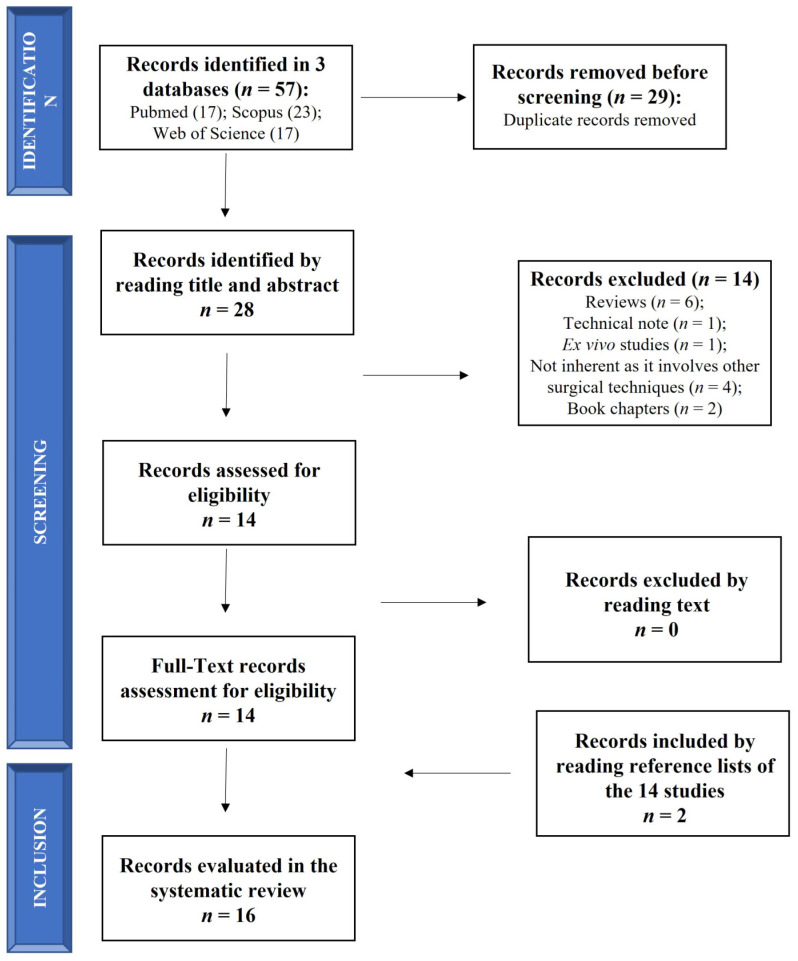
Schematic representation of studies search.

The search was performed on 1 November 2022 (from 1 November 2012 to 1 November 2022) according to Preferred Reporting Items for Systematic Reviews and Meta-Analyses (PRISMA) statement (Figure 1). The search was carried out on 3 electronic databases (PubMed, Scopus and Web of Science) to identify relevant papers using the following keywords with boolean operators: “(Retrograde drilling OR transtalar drilling) AND (osteochondral lesion of the ankle)”. The limits identified were (1) in PubMed: (i) language (English); (ii) publication date (from 1 November 2012 to 1 November 2022); (2) in Scopus and Web of Science: (i) language (English); (ii) publication date (between 2012 and 2022).

Relevant articles were screened using the title and abstract by 2 authors (FV and MM), and articles that did not meet the inclusion criteria were excluded. Only the clinical studies evaluating RD in OCL of the talus were included in this review and submitted to a public reference manager to eliminate duplicates and manage the references.

### 2.3. Information Extracted from Articles

The included full-text articles were retrieved and reviewed by the 2 authors (FV and MM), and any disagreement was resolved through discussion until a consensus was reached or with the involvement of a third author (GG). The following information was extracted from each paper and finally tabulated in Table 1 to summarize the evidence reported in each study: (a) References (Ref.); (b) study type; (c) complications; (d) grade/localization of lesion; (e) surgical procedures; (f) Follow-up (FU); (g) evaluations; (h) main results.

## 3. Results

### 3.1. Search Results

The initial literature search retrieved 17 studies from PubMed, 23 from Scopus and 17 from Web of Science for a total of 57 articles. After removing duplicates (29 papers) using a public reference manager (Mendeley Desktop 1.19.8) software, 28 papers remained. Among these 28 papers, 14 papers were excluded because they were reviews (*n* = 6), a technical note (*n* = 1), an ex vivo study (*n* = 1), non-inherent studies that involved other surgical techniques (*n* = 4) and book chapters (*n* = 2). The remaining 14 articles were reviewed and considered eligible. Two additional studies were found by reading the selected articles’ reference lists, so 16 studies were included in this systematic review in agreement with the PICO question and PRISMA methodological tool (Figure 1). 

Table 1 and Table 2 summarize the highlights of the studies and the characteristics of the patients. Among the 16 studies included in the present systematic review, 9/16 studies (56%) performed only the RD technique, compared or not to other surgical techniques [19,20,21,22,23,24,25,26,27], while 7/16 (44%) performed RD procedures with the addition of bone substitutes or bone marrow-derived cells (BMDC) [28,29,30,31,32,33,34].

#### 3.1.1. Main Results of RD Technique 

The main results were evaluated through clinical scores. The most used instruments for measuring the outcome of treatment in patients, who sustained a complex ankle or hindfoot injury, combined a clinician-reported and a patient-reported part, measuring pain and ankle instability, cartilage lesion grades, quality of life and activity in daily life or sports:

- American Orthopaedic Foot and Ankle Society (AOFAS) [19,20,21,24,25];
- Visual Analogue Score (VAS) [20,24,26];
- Japanese Society for Surgery of the Foot (JSSF) scale [23];
- Ankle activity score [21];
- International Cartilage Repair Society (ICRS) grade [21,27];
- Saxena criteria [25];
- Short Form-12 (SF-12) [26];
- Tegner score [26];
- Marx activity scores [26];
- Naal Sports inventory [26];
- Foot and Ankle Disability Index (FADI) [26].

As for imaging diagnostics, the approaches reported were magnetic resonance imaging (MRI) [19,20,21,22,24,27], computed tomography (CT) scans [20,22,23] and radiography [20]. For each study, the grades of OCL were indicated in Table 2 according to radiographic or MRI grading systems.

**Table 2:** Grading scores employed to classify lesion grades.

Grading Score Description		Grade	Ref.
Radiographic grading system	Pritsch Classification	II and III	[23]
		I	[24]
		I–III	[29]
		II and III	[33]
	Berndt and Harty clinical grade	I and II	[20]
		I–IV	[31]
MRI grading score	Anderson classification	II and III	[21]
		IIA	[22]
	Nelson classification system	I	[24]
	Hepple grade	I–III	[34]

In three studies, one case report [19] and two retrospective case series [20,21], 1 [19], six [20] and eight [21] patients, respectively, were treated with arthroscopic fluoroscopy-guided RD for ankle osteochondritis dissecans (OCD) of the talar head (14 mm in diameter) [19], posteromedial and central OCD [21], or posteromedial and central OCL of the talus [21]. In adolescent patients, one male of 14 years old [19], one female and five males of a mean of 13 years old [20] and three females and five males of a mean age of 14.9 years [21], AOFAS score improved during 24, 37 and 60 months of follow-up [19,20,21]. In addition, a return to the previous sport level was observed within 9 months, with symptom-free recovery at 12 months and SB healing [19]. Complete healing was observed in 50% of patients, with reduced VAS scores and 100% satisfaction [20]. Finally, Ikuta et al. showed that all patients return to their previous sport level within 6 months, with a 62.5% of good congruity of cartilage and a reduction of bone marrow lesions (BML) [21].

MRI, CT and radiography showed variable results, with evidence of SB healing with only some irregularities at the joint levels [19], complete healing in half of the patients [20] and good fragment incorporation, good cartilage congruity and reduction of BML [21]. 

In a case report [22] and a retrospective case series [23], OCL of the talus was treated with RD in association with synovectomy in one male of 53 years old [22] or with lateral ankle ligament repair or drilling for os subtibiale in two females and four males’ children of a mean age of 11.1 years [23]. 

Jeong et al. observed that even if cartilage depression gradually increased during 60 months of follow-up, no pain was registered after 12 months [22]. After 60 months, SB sclerosis and osteophyte formation, multiple cysts and BME were observed [22]. On the other hand, Minokawa et al. showed that the JSSF scale improved with good healing in 50% of patients after a mean of 22.8 months [23]. No degenerative changes were noted [23].

In the case series [24] and prospective case series [25], 16 young patients with a mean of 25 years of age, were affected by chronic lateral ankle instability (CLAI) with SB lesions of the talus in the medial position [24], and 32 patients with a mean of 32 years old suffered of symptomatic medial or lateral talus OCL [25]. In the study by Yasui et al., RD was associated with anterior talofibular ligament (ATFL) repair with modified Brostrom technique or ATFL reconstruction with autologous gracilis tendon [24], while Abd-Ella et al., performed anterior ankle arthroplasty with simultaneous modified Brostrom procedures for CLAI or RD [25]. After a mean of 26 and 29 months, AOFAS pain and function improved [24,25], VAS pain reduced, as well as the mean lesion area [24], with excellent results in 46.9% of patients and very satisfaction in 50% of patients [25]. MRI images showed that the mean lesion area decreased over time [24]. 

Finally, two studies compared the RD technique with other ones in 57 (21 females and 36 males) patients with a mean age of 37.1 years [26] and in 27 (17 females and 10 males) patients with a mean age of 16.9 years [28] affected by medial or lateral OCL of the talus [26,27]. Schwartz et al. compared RD with AD or microfracture for small lesions with cartilage loss, osteochondral autograft transfer (OAT) in larger lesions with subchondral plate defects and allograft cartilage implantation in uncontained defects [26]. After a mean of 79.9 months, in all the procedures, patient satisfaction was 77.2%, the FADI-sport score was 45.8, the Marx activity scale was 2.8, SF-12/PCS was 44, SF-12/MCS was 56.3, the Tegner score decreased and 85.7% of patients participated in some sport activities. However, RD showed the highest VAS pain, lowest VAS function and SF-12/MCS [26].

Korner et al. compared RD with bone marrow stimulation (BMS) and/or RD, flake fixation or autologous cartilage implantation (ACI) followed by autologous bone graft (ABG) implantation. The primary outcome was re-operation, showing that, after a mean of 42 months of follow-up, 25.9% of the patients underwent re-operation. Among them, the highest percentage of re-operation was observed for RD procedures. Re-operated patients had higher cartilage damage and lower ICRS stage than no re-operated ones [27]. In addition, MRI showed that in re-operated patients MOCART score was slightly higher than non-reoperated ones [27].

#### 3.1.2. Complications

No complications relating to RD techniques were reported in two studies [19,24], and no complications were found in the other three studies [20,21,23]. In one study, patients’ pain was high (VAS = 9), and SB sclerosis, osteophyte formation, cystic lesions, BME and thin articular cartilage were observed 60 months after the RD procedure [22]. Unsatisfaction, with associated persistent pain and no improvement after 12 months, was experienced by one patient [25], or secondary surgeries were required [26].

#### 3.1.3. Main Results of RD Technique Associated with Bone Substitutes or Cells

The main scores employed were the same as those from the previous studies, such as AOFAS [28,29,30,33,34], FADI and VAS [29]. The other scores evaluated (1) the impact that foot pathologies have on the patient’s perceived state of health in terms of pain, disability and activity limitations, such as foot function index (FFI) [28], (2) subjective and objective assessment of pain and discomfort, such as Roles and Maudsley (RM) scores [30], (3) the return to activity (RTA) [30] and the return to sport survey [31]. Finally, the Foot and Ankle Outcome Score (FAOS) [33] considers pain, other symptoms, activities of daily living, sport and recreational function and foot- and ankle-related quality of life. In addition, radiography [28,31,32,33] and MRI [28,29,32,33,34] were used to evaluate the results and the lesion grades, as indicated in Table 2.

After RD procedures, ABG was used to fill the tibia OCL of the talar dome and OCD of the ankle [28,29,30,31]. Perera et al. treated one male of 46 years old with arthroscopic debridement, cheilectomy and microfracture, followed, after 6 months, by arthroscopic debridement defect curettage, RD and ABG. No pain was observed after 6 weeks, and AOFAS increased from 3 to 12 months with MOXFG of 46 and FFI of 17. The complete integration of the graft and overlying cartilage was observed, and clinical improvements were maintained for 36 months [28]. Fluoroscopy-guided RD with ABG was employed in 38 patients (16 females and 22 males) with a mean age of 33.2 years. During a mean follow-up of 29 months, AOFAS pain and function improved, and VAS pain and VAS function scores were respectively reduced and increased, with 85% of satisfaction and 12.2% of complete bone remodeling, showing that grade I and II lesions had better results than grade III ones [29]. Saxena et al. treated small OCL lesions without intact cartilage with microfracture and PRP, while those with intact cartilage with RD, ABG and PRP in 204 patients (85 females and 119 males) with a mean age of 37.9 and 39.7 years for females and males, respectively. After a mean of 82.5 months, the RTA was 7.9, the RM score was 1.3 and AOFAS increased [30]. Finally, Kramer et al. performed RD with a bioabsorbable implant or ABG in 100 patients (75 females and 25 males) with a mean age of 14.3 years. After a mean of 39.6 months, lesions improved in 64% of cases, satisfaction was 81.8%, the rate of return to sport after 6 months was 84.1% and FAOS was 77 [31].

In two studies, RD was followed by the implantation of a biodegradable orthopedic biocomposite (composed of calcium sulfate and/or calcium phosphate) with [32] or without recombinant human bone morphogenetic protein 2 (rhBMP2) [33]. Two males, 44 and 31 years old, showed an OCL consolidation after 17 weeks and clinical and radiographic improvement after 2 months [32]. In seven patients (four females and three males) of a mean of 36 years old, after a mean of 29 months, AOFAS total score and FADI increased, AOFAS pain decreased, and good restoration of the medial talar dome contour, bony ingrowth and remodeling of the lesion were shown [33].

Finally, Gao et al. treated 69 patients (32 females and 37 males; mean age 46.2 years) affected by talus OCL with an injection of BMDC after RD, with or without focused extracorporeal shock wave treatment (ESWT) applied after the injection. After a mean of 49.2 months, this procedure increased AOFAS, daily life function and the regression of the lesion. The use of ESWT increased AOFAS pain and function and the reduction of lesions more than the absence of ESWT. In addition, lesions of grades I and II showed significantly better results than those of grade III [34]. 

#### 3.1.4. Complications

One study did not report complications [28]. In the studies that employed ABG, ankle swelling for up to 3 months, minor hypesthesia of the forefoot and delayed superficial wound healing in 13.2%, 5.3% and 2.6% of cases [29], hardware removal (3.4%) and revision surgery (2%) [30] and re-operation after a mean of 20.4 months in 26.6% of cases [31] were reported. Achilles tendon pain and symptomatic subsidence after 17 weeks in one patient and minor anterior tibial spur removal (28.6%) and partial synovectomy (71.4%) were shown with the use of calcium sulfate and/or calcium phosphate biocomposite [32,33]. Finally, ankle swelling for 12 months was observed in 12.2% of patients treated with BMDC and ESWT and in 25% of patients that did not use ESWT, while hypesthesia of the midfoot in 2.4% of patients with BMDC alone [34]. 

### 3.2. Association between Main Results and Gender, BMI or Age

Table 3 summarizes the gender, age and BMI of patients of the studies and, as observed in Table 4, in most of the studies (75%), the associations between main results and gender, BMI or age were not evaluated [19,20,21,22,23,24,25,26,28,32,33,34]. Andersen et al. showed that gender, BMI and age did not influence the outcomes of the surgical procedure [29]. Similarly, another study did not observe a significant association between re-operated patients and gender, BMI and age [27]. One study did not find significant differences between males and females as regards mean RTA (*p* = 0.08), postoperative AOFAS (*p* = 0.52) and post-RM score (*p* = 0.41). The association between BMI and age with outcomes was not evaluated [30]. Only one study showed that females had worse FAOS than males (*p* < 0.01), and a BMI over 30 induced worse FAOS than BMIs of 16–24 and 25–30, even if no association was evaluated between FAOS and age [31].

## 4. Discussion

The literature analysis performed in the present systematic review returned a heterogeneous scenario of clinical applications of RD technique for treating talus OCL. In 10 years of published literature, 16 clinical studies were obtained and discussed, and several differences were found regarding the types of study, follow-up, number and age of patients, lesion type, dimensions and grade and comparison groups. 

Most of the included studies were retrospective (44%), three were case reports (19%) [19,22,28], two were prospective (12%) [25,33], one was a case series (6%) [24] and three studies did not specify the typology (19%) [27,29,35]. In nine studies, the RD technique was performed alone without the addition of bone substitutes [19,20,21,22,23,24,25,26,27], while seven studies filled talus lesions with ABG [28,29,31], ABG added with PRP [30], biodegradable calcium sulfate/calcium phosphate biocomposites [32,33] and BMDC with or without physical stimulation [34]. 

The follow-up varied among the studies, ranging from a minimum of 1 week [32] to a mean of 7 years [26,30]. The other studies have interim follow-ups of 2 [21,23,24,25,29,33], 3 [20,27,28,31], 4 [34] and 5 [19,22] years.

**Figure 2:**
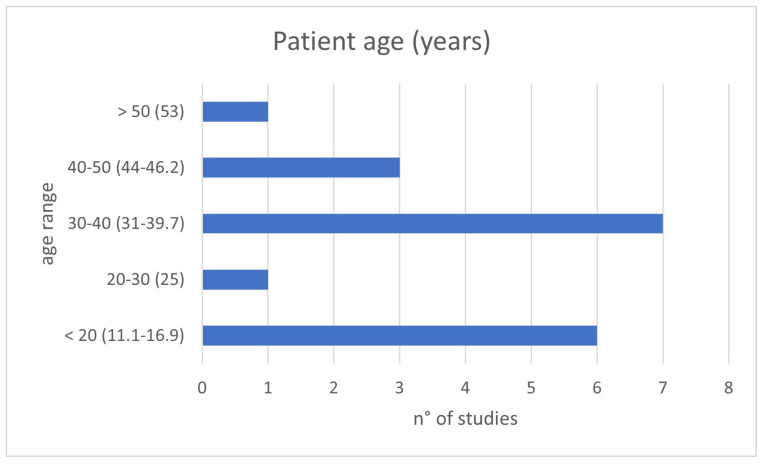
Patient ages of the included studies divided by age range: <20, 20–30, 30–40, 40–50 and >50 years old.

The clinical relevance of the topic is corroborated by the young age at which patients were present for treatment: in about 80% of evaluated studies, patients’ age was under 40 years old, and just under half involved pediatric patients (Figure 2). The presentation is often with painful symptoms, which therefore require an approach that is as decisive as possible and which allows them to resume daily activities as expected for a young adult or to address skeletal development in developing-age patients.

**Figure 3:**
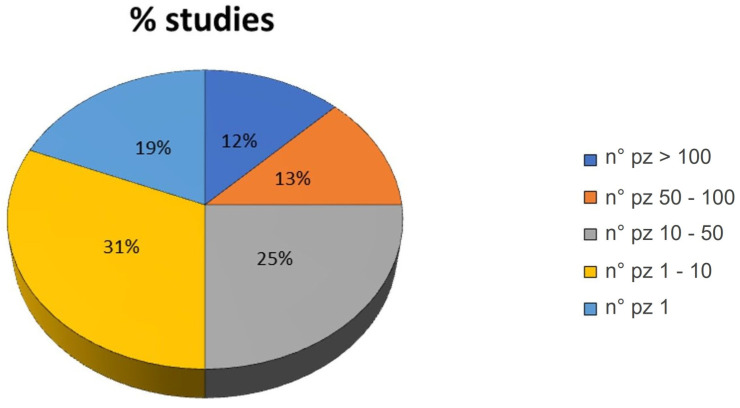
Pie chart of the percentage of studies grouped by the number (n°) of patients involved in the study: >100, 50–100, 10–50, 1–10 and 1.

Also, the number of patients treated in each study varied from a minimum of one patient (19% of the studies) to a maximum of >100 patients (12% of the studies). As observed in Figure 3, most of the studies enrolled 1–10 patients (31% of the studies).

Lesion dimensions were not always reported, while the position in the talus was usually specified; in most cases, these were posteromedial or medial lesions [20,21,23,24,25,26,27,29,30,32,33,34]; some cases involved further surgery following a previous one which had not improved the patient’s clinical condition. 

Dimensions of the lesions, when reported, were around 125–150 mm^2^ [19,25,26,27,30], or <125 mm^2^ [27,30,31,33,34] and only one study treated lesions > 1500 mm^2^ [30]. The authors did not find a correlation between lesion dimensions and the outcomes. Almost all the studies used the same treatment for any type of lesion size, while two studies treated the lesions differently based on the size. More precisely, Korner et al. treated lesions < 150 mm^2^ with AD and/or microfracture and lesions > 150 mm^2^ with OAT [26], Saxena et al., employed arthrotomy, microfracture and PRP in lesions of 125 mm^2^, osteotomy, curettage, ABG and PRP in 125–1500 mm^2^ lesions, and allograft, PRP and fixation in >1500 mm^2^ lesions [30]. All these authors found that the treatments performed in the smaller lesions showed higher improvement, as regards pain, activity level and patient satisfaction, probably due to the small dimensions of the lesions. 

Heterogeneous classification systems were reported to classify the grade of the lesions: the Pritsch Classification System [23,24,29,33], Berndt and Harty stage [20,31], Anderson classification [21,22], Nelson classification system [24] and Hepple Grade [34]. As reported in Table 5, they are radiographic or MRI grading systems and consider more or less the same parameters. Some scores are more oriented towards the evaluation of cartilage and SB (Pritsch Classification, Nelson classification system and Hepple grade), and the others of only SB (Berndt and Harty clinical grade and Anderson classification). 

The authors that applied the first group of scores [23,24,29,33] treated lesions with fraying or fibrillated cartilage [23,24,29,33,34] and with bone fragments that had detached and remained in the defect [23,29,33] and no lesions of grade IV or V were treated. The studies that employed the second group of scores [20,21,22,31] treated OCL with localized SB compression [20], separated bone fragments [20,21], undisplaced bone fragments [21], SB cysts [22] and only in one study lesions of all grades were treated [31]. However, all the studies found that the RD technique improved all lesion types, although the initial lesion grade of the defect, for the most part, never exceeded grade 3. 

However, the differences in the location and grade of lesions in the present studies make it difficult to uniformly compare studies based on lesion grades. 

Regarding comparison groups of treatment, some studies performed only RD in all patients without comparing different techniques or treatments [19,20,21,22,29,33], allowing us to monitor the success of the RD technique during the follow-up. All these studies showed increasingly positive results up to several months after RD. 

One study that compared RD with other techniques, such as AD, OAT and the use of allografts, observed that RD reduced VAS pain and improved VAS function and SF-12/MCS score to a lesser extent than other techniques [26]. Similarly, in another study, the use of allograft remained the best treatment [30]. Finally, one study compared the results of two patient groups treated with RD, and BMDC was stimulated or not with ESWT. It was observed that stimulation improved AOFAS pain and function and reduced the lesion area more than the not-stimulated one [34]. The other studies, although having different groups of patients treated with different techniques, reported the results in their entirety without highlighting differences between groups [23,24,25,27,28,31,32], making it difficult to compare RD with other treatments.

Gender and BMI correlation to the outcomes and complications remain underestimated and critical aspects.

The gender-related response to treatments, when indicated, showed very close numbers between males and females who undergo this surgery. Overall, considering all studies, the numbers of females and males were 277 ± 28.15 and 301 ± 29.42, respectively, without significant differences. 

In the literature, there is a growing awareness of the difference between gender in talus OCL presentation and in the outcomes from treatments such as autologous osteochondral transplantation or BMS [40,41]. It might be of great interest to differentiate the results obtained from the reported studies based on patient gender to reveal any difference in the clinical presentation or the results or to eventually highlight the comparable effectiveness of RD in the outcomes regardless of gender.

In the present review, most studies did not evaluate the association between clinical outcomes and gender [19,20,21,22,23,24,25,26,28,32,33,34]. In two studies, the authors observed no differences between males and females as regards clinical scores [29,30] or re-operation rate [27]. Only one study underlined that females had worse FAOS than males [31]. 

Similarly, the correlation between the BMI and age of the patients was investigated. Furthermore, in this case, most studies did not evaluate this aspect [19,20,21,22,23,24,25,26,28,32,32,33,34], while one study showed that BMI > 30 induced worse FAOS outcomes than 16–24 and 25–30 BMI [31].

Another critical aspect is related to complications, which were not always reported or described in detail [19,20,21,23,24,28], making comparing study results difficult and generally complicating the global evaluation of the treatment outcomes. When reported, complications mainly regarded persistent pain [22,27,32], presence of SB sclerosis, osteophyte formation, cystic lesions and BME [22] and no improvement after 1 year from treatment [25]. Re-operation was a complication of some studies [26,27,30,31], and other minor complications regarded ankle swelling and hypesthesia of the forefoot [29,34], delayed superficial wound healing [29], minor anterior tibial spur removal and partial synovectomy [33].

On the other hand, alongside all the aforementioned heterogeneous aspects in the studies, instead, there was a great uniformity in the choice of diagnostic and monitoring tools (mainly radiological imaging, MRI or CT scan) [19,20,21,22,23,24,27,28,29,31,32,33,34], as well as in the choice of clinical scores to be applied, among which the most common remain the VAS [20,24,26,29] and AOFAS [19,20,21,24,25,28,29,30,33,34], which allows the easy and direct comparison of patient’s outcomes.

However, despite the scarcity of works and the heterogeneity of several different aspects of the included studies, the results tended to be positive. In fact, it was usually observed a good rate of satisfaction from patients and improvement in clinical scores, with a reduction of pain and a return to daily activities and sports at 3–12 months from surgery; imaging investigations showed new bone formation and, when present, integration of grafts employed during surgery. 

## 5. Conclusions

To conclude, RD has proved to be an advantageous technique in situations where an osteochondral defect of the talus still has the superficial cartilage intact. Although it is a long-standing surgical technique, introduced in 1981, there are still few clinical data produced in the last 10 years in that regard. This systematic review showed the most employed clinical scores and treatments performed in literature to treat OCL with RD technique, alone or in combination with cells, other bone substitutes, or other surgical techniques. In addition, it underlines that lesions on which RD shows the best results are of I-III grades and do not exceed 150 mm^2^ in size.

Future studies are necessary to investigate which patient and lesion characteristics are associated with persistent symptoms that eventually require surgical intervention. The clinical studies analyzed in this review are different in terms of type, number and age of patients treated, follow-up and patient comparison groups, making it difficult to draw conclusions. Further clinical or preclinical studies are mandatory to underline the success of this technique, especially related to gender differences if they exist. Gender differences are still a debated topic in the literature for a variety of musculoskeletal diseases, indicating the necessity to perform more preclinical and clinical studies to elucidate the gender-based determinants and mechanisms at the base of these pathologies, also in the view of developing gender-specific protocols and tailored drugs.

## Figures and Tables

**Table 1:** Summary table of the main findings from the studies included for review.

Ref.	Study Type	Complications	Grade/Localization of Lesion	Surgical Procedure	Fu (mo)	Evaluations	Main Results
Corominas 2016 [19]	Case report	n.r.	OCD of the talar head (153 mm^2^)	Arthroscopic fluoroscopy-guided RD	>60	AOFAS MRI	At 9 mo: return to former competitive level. At 12 mo: symptom-free. At 24 mo: AOFAS = 90. At 60 mo: AOFAS = 100; MRI evidence of SB healing, with some irregularities at the joint level and a very well-preserved joint space.
Masquijo 2016 [20]	Retrospective chart review	No complications	OCD of the talus: Posteromedial (*n* = 5); Central (*n* = 1). Berndt and Harty stage: Grade I (*n* = 5) and II (*n* = 1)	Arthroscopic fluoroscopy-guided RD	Mean 37 (16–69)	AOFAS VAS Radiography MRI CT	Progression toward healing Asymptomatic pz Complete healing (50%) ↑ AOFAS ↓ VAS Satisfaction (100%) 3/6 pz complete radiographic healing at the last follow-up
Ikuta 2020 [21]	Retrospective case series	No complications	Symptomatic stable OCD of the talus: Posteromedial (*n* = 7); Central (*n* = 1) Anderson classification: Grade II (*n* = 3) and III (*n* = 5)	Arthroscopic fluoroscopy-guided RD	Mean 24 (12–60)	AOFAS Ankle activity score ICRS grade MRI	↑ AOFAS score and ankle activity score. At 6 mo: return to former competitive level. ICRS grade 0 = 37.5% ICRS grade I = 62.5% No signs of fragment instability or subsidence. Good fragment incorporation in 6 pz and fair in 2 patients. Good cartilage congruity in 5 pz and slight irregularity with intact cartilage in 3 pz. BML areas ↓, but still detectable at 1 yr.
Jeong 2016 [22]	Case report	Pain (VAS = 9) at 60 mo Subchondral sclerosis and osteophyte formation, cystic lesions, bone marrow edema in talus, thin talar articular cartilage at 60 mo	OCL of the talus with a central subchondral cyst, multiple medial small SB cysts and BME. Anderson classification: 2A	Arthroscopic synovectomy + RD	60	MRI CT	At 12 mo: ↑ depression of the cartilage and no pain. At 60 mo: SB sclerosis and osteophyte formation, multiple cystic lesions, BME in talus, thin talar articular cartilage.
Minokawa 2020 [23]	Retrospective case series	No complications	OCL of the talus: Posteromedial (*n* = 8); Pritsch Classification System: Grade II (*n* = 7) and III (*n* = 1)	RD (*n* = 6); RD + arthroscopic lateral ankle ligament repair (*n* = 1); RD + drilling for os subtibiale (*n* = 1)	Mean 22.8 (8–51)	JSSF scale CT	↑ JSSF scale. CT healing: good (50%), fair (37.5%), poor (12.5%). No degenerative changes were noted in the radiographs at the final follow-up.
Yasui 2014 [24]	Case series	n.r.	CLAI with SB lesion of the talus: Medial (*n* = 16). Nelson classification system: Grade I (*n* = 16). Modified Pritsch classification: Grade I (*n* = 16)	Fluoroscopic guidance RD + ATFL repair with the modified Broström technique (*n* = 8); Fluoroscopic guidance RD + ATFL reconstruction with autologous gracilis tendon (*n* = 8)	Mean 29 (24–46)	AOFAS VAS MRI	↑ mean AOFAS (pain and function). ↓ VAS pain and mean lesion area. Mean lesion area ↓.
Abd_Ella 2017 [25]	Prospective case series	Unsatisfaction with persistent pain and no improvement for 1 yr (1 pz)	Small OCL of the talus (area < 150 mm^2^, cyst depth < 7 mm): Medial (*n* = 29); Lateral (*n* = 3)	Anterior ankle arthroscopy + modified Brostrom procedures for lateral ankle instability (4/32 pz); Anterior ankle arthroscopy + fluoroscopy-guided RD (5/32 pz)	26 (12–48)	AOFAS Saxena criteria	↑AOFAS Results: Excellent (46.9%), good (37.5%), fair (15.6%). Very satisfied (50%), satisfied (28.1%), satisfied with reservations (18.8%), unsatisfied (3.1%).
Schwartz 2021 [26]	Retrospective series	Secondary procedure (14.0%): AD (62.5%); RD (12.5%); OAT (12.5%); Allograft (12.5%)	OCL of talus (125.2 mm^2^): Medial (71.9%); Lateral (28.1%)	Lesions with isolated cartilage loss < 150 mm^2^: AD and/or microfracture (*n* = 32); Intact cartilage cap with a deficient SB plate: RD (*n* = 7); Lesions > 150 mm^2^ or with underlying SB plate defects: OAT (*n* = 10); Uncontained defects: allograft cartilage implantation (*n* = 8)	Mean 79.9 (17–209.8)	VAS SF-12 FADI-sports; Tegner score; Marx activity scores; Naal Sports inventory; Pz satisfaction	Satisfaction = 77.2%. FADI-Sport score = 45.8. Marx activity scale = 2.8. SF-12/PCS = 44.0. SF-12/MCS = 56.3. ↓ Tegner score. AD, OAT, Allograft: ↓ VAS pain; ↑ VAS function Allograft, followed by AD, OAT and RD: ↑ SF-12/MCS.
Korner 2021 [27]	/	Re-operation (25.9%): Arthroscopy + BMS (28.6%); Arthroscopy + RD (57.1%); ACI + ABG (14.3%)	OCL of the talus: Medial (mean 127 mm^2^) (*n* = 17); Lateral (mean 104 mm^2^) *n* = 10)	Arthroscopy + BMS (*n* = 8); Arthroscopy + RD (*n* = 8)); Arthroscopy + BMS + RD (*n* = 1); Arthrotomy + flake fixation (*n* = 1); ACI + ABG (*n* = 9)	42 (6–117)	ICRS grade MOCART score MRI	In re-operated pz: ↑ advanced cartilage damage; ↓ ICRS stage than non-reoperated pz. In re-operated pz: ↑ MOCART score than non-reoperated pz.
Perera 2015 [28]	Case report	n.r.	OCL of the tibia	Step 1): Arthroscopic debridement + cheilectomy + microfracture; Step 2) (after 6 mo): Arthroscopic debridement + defect curetted + RD + ABG	2, 6, 12 and 16 wks, 36 mo	Radiography MRI AOFAS MOXFQ FFI	At 6 wks: No pain, AOFAS score = 58. At 12 mo: AOFAS score = 86, MOXFG = 46, FFI = 17 At 16 wks: complete integration of the graft with maintained overlying articular cartilage. At 36 mo: improvements maintained at 36 mo.
Anders 2012 [29]	/	Ankle swelling up to 3 mo (13.2%); Minor hypesthesia of the forefoot (5.3%); Delayed superficial wound healing (2.6%)	Undisplaced OCL of the talus (7–14 mm): Medial (*n* = 36); Apical (*n* = 29); Lateral (*n* = 4); Central (*n* = 1) Pritsch classification Grade I (*n* = 12), II (*n* = 22) and III (*n* = 7)	Fluoroscopy-guided RD + ABG	29 ± 13 (12–54)	AOFAS VAS MRI	↑ AOFAS and VAS function. ↓VAS pain. 85% satisfaction. Grade I and II lesions: ↑ results than grade III lesions. Complete bone remodeling (12.2%).
Saxena 2022 [30]	/	Hardware removal (3.4%), Revision surgery (2%)	OCL of the talar dome “small” (<125 mm^2^), “medium” (125–1500 mm^2^) and “large” (≥1500 mm^2^): Antero-lateral (*n* = 55); Antero-medial (*n* = 54); Central-lateral (*n* = 11); Central-medial (*n* = 27); Medial-central (*n* = 6); Postero-medial (*n* = 30); Postero-lateral (*n* = 22).	Small lesions without intact cartilage: arthrotomy + microfracture + PRP (*n* = 112); Lesions with small subchondral cysts and intact cartilage: RD (*n* = 31); Lesions with large subchondral defect: RD + ABG + PRP (*n* = 8); Medium lesions: osteotomy + curettage + ABG + PRP (*n* = 60); Large lesion: allograft + PRP + fixation (*n* = 7)	Mean 82.5 ± 34.6 (24–132)	AOFAS RM score RTA	RTA = 7.9 ± 5 mo. ↑ AOFAS score. RM score = 1.3± 0.5. Allograft, autograft and osteotomy: ↓ AOFAS. Allograft: ↓ activity level.
Kramer 2015 [31]	Retrospective chart review	Re-operation at 20.4 mo (26.6%): Lesions with no change or worse ↑ re-operation rate than lesions healed or improved	OCL of the ankle (113 ± 62 mm^2^): Medial (*n* = 80); Lateral (*n* = 22); Central (*n* = 5); Tibia (*n* = 2). Berndt and Harty stage: Grade I (*n* = 14), II (*n* = 50), III (*n* = 16) and IV (*n* = 3)	Fluoroscopy or not guided RD (*n* = 59); RD + internal fixation with a bioabsorbable implant (*n* = 22); Excision of lesion + microfracture (*n* = 27); RD + ABG (*n* = 1)	Mean 39.6 (12–129.6)	Radiography Return to sport survey FAOS	Results: Poor (30.3%), fair (21.1%) and good (48.6%). Lesion: healed (16%), improved (64%), unchanged (18%) and worse (3%). Satisfaction (81.8%). At 6 mo: Return to sport (84.1%). FAOS = 77 ± 18.
Mehta 2012 [32]	Retrospective review	Case 1: Achilles tendon pain and symptomatic subsidence at 17 wks. Case 2: No complications	OCL of the talus: Posteromedial lesion of grade I (Case 1); Posteromedial lesion (1 cm) with BME and with grade III (Case 2)	Case 1: RD + rhBMP-2 + PRO-DENSE regenerative graft; Case 2: RD + rhBMP-2 + Hydroset	1 wk–18 mo	Radiography MRI	Case 1: ↑ early clinical and radiographic findings. At 17 wks: Consolidation of OCL. Case 2: At 1 mo: ankle motor strength = 5/5, ↑ radiographic appearance. At 4 mo: radiographic resolution of the OCL, new bone formation, return to work.
Beck 2015 [33]	Prospective case series	Minor anterior tibial spur removed (28.6%); Partial synovectomy (71.4%)	Undetached OCL of the medial talar dome (≥ 100 mm²). Pritsch classification: Grade II (*n* = 5) and II-III (*n* = 2)	Arthroscopically-guided RD + CaSO4-CaPO4 bone graft substitute	24.1 (20–28)	AOFAS FADI score Radiography MRI	↑AOFAS and FADI scores. ↓AOFAS pain score. Good restoration of the medial talar dome contour. Bony ingrowth and remodeling of the lesion. Disappearing of bone bruising adjacent to the lesion.
Gao 2017 [34]	Retrospective, non-blinded comparative study	Ankle swelling for 12 mo (Group A: 12.2%; Group B: 25%) Hypesthesia of the midfoot (Group B: 2.4%)	Unilateral undisplaced OCL of the talus (mean 110 mm^2^, 70–140 mm^2^): Medial (*n* = 61); Lateral (*n* = 8). Group A (*n* = 41) and Group B (*n* = 28). Hepple grade: Grade I (*n* = 19), II (*n* = 42) and III (*n* = 8)	Group A (*n* = 41): RCD + BMDC + ESWT Group B (*n* = 28): RCD + BMDC	Mean 49.2 ± 33.6 Group A: 44.4 ± 14.4 Group B: 51.6 ± 26.4	AOFAS MRI	Groups A, B: ↑ overall AOFAS, pain relief, function, daily life function, progressive regression of the lesion. Group A: ↑ AOFAS pain and function, incidence of distinct lesion reduction than group B. Grade I and II: ↑ results than grade III.

Abbreviations: ABG = autologous bone graft; ACI = autologous chondrocyte implantation; AD = anterograde drilling; AOFAS = American Orthopaedic Foot and Ankle Society; ATFL = anterior talofibular ligament; BMDC = bone marrow-derived cells; BML = bone marrow lesion; BME = bone marrow edema; BMS = bone marrow stimulation; CaSO4-CaPO4 = calcium sulfate-calcium phosphate; CLAI = chronic lateral ankle instability; CT = computed tomography; ESWT = extracorporeal shock wave treatment; FADI = Foot and Ankle Disability Index; FAOS = Foot and Ankle Outcome Score; FFI = foot function index; FU = follow-up; ICRS = International Cartilage Repair Society; JSSF = Japanese Society for Surgery of the Foot; mo = months; MOCART = Magnetic Resonance Observation of Cartilage Repair Tissue; MOXFQ = Manchester–Oxford Foot Questionnaire; MRI = magnetic resonance imaging; n.a. = not applicable; n.r. = not reported; OAT = osteochondral autograft transfer; OCD = osteochondritis dissecans; OCL = osteochondral lesions; PRP = platelet rich plasma; pz = patients; RCD = retrograde core drilling; RD = retrograde drilling; Ref. = references; rhBMP-2 = recombinant human bone morphogenetic protein-2; RM = Roles and Maudsley; RTA = return to activity; SB = subchondral bone; SF-12/MCS = Short Form-12/mental component score; SF-12/PCS = Short Form-12/Physical Component Summary; VAS = Visual Analogue Score; wks = weeks.

**Table 3:** Gender, age and BMI of patients of the studies included in the review.

Ref.	Pz (n°) (F vs. M)	Age (yrs)	BMI (Kg/m^2^)
Corominas 2016 [19]	1 M	14	n.r.
Masquijo 2016 [20]	6 (1 F, 5 M)	Mean 13 (11–15)	n.r.
Ikuta 2020 [21]	8 (3 F, 5 M)	Mean 14.9 (11–19)	20.0 (17.2–23.9)
Jeong 2016 [22]	1 M	53	23.6
Minokawa 2020 [23]	6 (2 F, 4 M)	Mean 11.1 (9–12)	19.2 (15.6–31.0)
Yasui 2014 [24]	16 (11 F, 5 M)	Mean 25 (14–49)	n.r.
Abd_Ella 2017 [25]	32 (10 F, 22 M)	Mean 32 ± 8 (18–50)	n.r.
Schwartz 2021 [26]	57 (21 F, 36 M)	Mean 37.1 (15–62)	27.7 (27.2–28.3)
Korner 2021 [27]	27 (17 F, 10 M)	Mean 16.9 ± 2.2	22.64 (18.0–39.3)
Perera 2015 [28]	1 M	46	n.r.
Anders 2012 [29]	38 (16 F, 22 M)	Mean 33.2 (11–56)	24.8 ± 3.6
Saxena 2022 [30]	204 (85 F, 119 M)	F mean 37.9 ± 17.4 (range 12–74); M mean 39.7 ± 15.2 (range 5–68)	n.r.
Kramer 2015 [31]	100 (75 F, 25 M)	Mean 14.3 (7–18)	23.6 ± 4.5 (16.6–38.9)
Mehta 2012 [32]	Case 1: 1 M; Case 2: 1 M	Case 1: 44 yrs; Case 2: 31 yrs	n.r.
Beck 2015 [33]	7 (4 F, 3 M)	Mean 36 (18–69)	n.r.
Gao 2017 [34]	69 (32 F, 37 M)	Mean 46.2 (19–62)	25.1 ± 4.9

BMI = body mass index; F = female; M = male; pz = patients.

**Table 4:** Correlation between main results and gender, BMI and age of the patients of the studies included in the review.

Ref	Gender	BMI	Age
Corominas 2016 [19]	n.a.	n.a.	n.a.
Masquijo 2016 [20]	n.r.	n.r.	n.r.
Ikuta 2020 [21]	n.r.	n.r.	n.r.
Jeong 2016 [22]	n.a.	n.a.	n.a.
Minokawa 2020 [23]	n.r.	n.r.	n.r.
Yasui 2014 [24]	n.r.	n.r.	n.r.
Abd_Ella 2017 [25]	n.r.	n.r.	n.r.
Schwartz 2021 [26]	n.r.	n.r.	n.r.
Korner 2021 [27]	Re-operated pz (males = 2 vs. females = 5); No re-operated pz (males = 8 vs. females = 12). No significant differences were detected for gender.	Re-operated pz (21.7 kg/m^2^); No re-operated pz (23.6 kg/m^2^). No significant differences were detected for BMI.	Re-operated pz (16.3 ± 1.6 yrs); No re-operated pz (17.1 ± 2.4 yrs). No significant differences were detected for age.
Perera 2015 [28]	n.a.	n.a.	n.a.
Anders 2012 [29]	No significant differences were detected for gender.	No significant differences were detected for BMI.	No significant differences were detected for age.
Saxena 2022 [30]	Mean RTA (males = 8.0 ± 4.9 mo vs. females = 7.8 ± 5.1 mo; *p* = 0.08); Postoperative AOFAS (males = 96 ± 3.3 vs. females = 96.3 ± 3.7; *p* = 0.52); Post-RM scores (males = 1.3 ± 0.5 vs. females = 1.2 ± 0.5; *p* = 0.41). No significant differences were detected for gender.	n.r.	n.r.
Kramer 2015 [31]	Total FAOS (males = 444 ± 44 vs. females = 368 ± 93; *p* < 0.01). Females showed worse FAOS than males.	BMI >30 showed worse FAOS outcomes than 16–24 and 25–30 BMI.	n.r.
Mehta 2012 [32]	n.a.	n.a.	n.a.
Beck 2015 [33]	n.r.	n.r.	n.r.
Gao 2017 [34]	n.r.	n.r.	n.r.

AOFAS = American Orthopaedic Foot and Ankle Society; BMI = body mass index; F = female; FAOS = Foot and Ankle Outcome Score; M = male; yrs = years; pz = patients; RM = Roles and Maudsley; RTA = return to activity.

**Table 5:** Description of the different grading scores employed to grade lesion types.

Grading Score	Description
Pritsch Classification (radiographic grading system) [36]	0 = normal cartilage with abnormal bone
	I = cartilage fibrillation
	II = fraying cartilage
	III = bone fragmentation detached remaining in the defect
	IV = bone fragment detached and loose
Berndt and Harty clinical grade (radiographic grading score) [37,38]	I = localized area of SB compression
	II = separated bone fragments
	III = undisplaced bone fragments
	IIIA = detached and rotated bone fragments
	IV = bone fragments displaced and inverted in its fracture bed
Anderson classification (MRI grading score) [38]	I = SB compression
	II = incomplete separation of bone fragment
	IIA = presence of subchondral cyst
	III = undisplaced bone fragment
	IV = displaced bone fragment
Nelson classification system (MRI grading score) [39]	0 = normal cartilage
	I = intact cartilage with soft changes
	II = high-signal breach of the cartilage
	III = thin, high-signal rim extending behind the osteochondral fragment
	IV = mixed- or low-signal loose body in the center of the lesion or free within the joint
Hepple grade (MRI grading score) [35]	I = only cartilage damage
	IIa = cartilage injury, SB fracture and bone edema
	IIb = cartilage injury, SB fracture without bone edema
	III = detached but undisplaced fragments
	IV = detached and displaced bone fragments
	V = detached and displaced bone fragments with SB cysts

## Data Availability

The data that support the findings of this study are available from the corresponding author upon reasonable request.
